# Interactive Visual Displays for Interpreting the Results of Clinical Trials: Formative Evaluation With Case Vignettes

**DOI:** 10.2196/10507

**Published:** 2018-06-25

**Authors:** Jiantao Bian, Charlene Weir, Prasad Unni, Damian Borbolla, Thomas Reese, Yik-Ki Jacob Wan, Guilherme Del Fiol

**Affiliations:** ^1^ Department of Biomedical Informatics University of Utah Salt Lake City, UT United States; ^2^ George E. Whalen Veterans Affairs Medical Center Salt Lake City, UT United States; ^3^ Intelligent Medical Objects Chicago, IL United States

**Keywords:** clinical decision-making, clinician information needs, information display, information foraging theory, information seeking behavior

## Abstract

**Background:**

At the point of care, evidence from randomized controlled trials (RCTs) is underutilized in helping clinicians meet their information needs.

**Objective:**

To design interactive visual displays to help clinicians interpret and compare the results of relevant RCTs for the management of a specific patient, and to conduct a formative evaluation with physicians comparing interactive visual versus narrative displays.

**Methods:**

We followed a user-centered and iterative design process succeeded by development of information display prototypes as a Web-based application. We then used a within-subjects design with 20 participants (8 attendings and 12 residents) to evaluate the *usability* and *problem-solving* impact of the information displays. We compared subjects’ perceptions of the interactive visual displays versus narrative abstracts.

**Results:**

The resulting interactive visual displays present RCT results side-by-side according to the Population, Intervention, Comparison, and Outcome (PICO) framework. Study participants completed 19 *usability* tasks in 3 to 11 seconds with a success rate of 78% to 100%. Participants favored the interactive visual displays over narrative abstracts according to *perceived efficiency, effectiveness, effort, user experience* and *preference* (all *P* values <.001).

**Conclusions:**

When interpreting and applying RCT findings to case vignettes, physicians preferred interactive graphical and PICO-framework-based information displays that enable direct comparison of the results from multiple RCTs compared to the traditional narrative and study-centered format. Future studies should investigate the use of interactive visual displays to support clinical decision making in care settings and their effect on clinician and patient outcomes.

## Introduction

### Background

At the point of care, clinicians have many clinical questions that they are unable to answer with the best available evidence [1]. Unanswered questions are missed opportunities to improve patient care decisions and for just-in-time learning [2]. Primary literature resources (eg, PubMed) contain answers to most of these questions [3], but their use at the point of care is still limited due to barriers such as lack of time and significant cognitive effort imposed by the evidence search and interpretation process [4,5].

Abstracts in scientific manuscripts are typically presented according to the well-established “background, introduction, methods, results, discussion, conclusion” structure [6]. However, this study-centered structure may not be optimal to support clinicians’ patient-centered conceptual models. The average time for clinicians to look up clinical questions on PubMed ranges from 5 to 60 minutes [7]. In addition, clinicians report high levels of dissatisfaction with their information seeking experience [8,9]. Ultimately, clinicians’ challenges in consuming evidence from the primary literature may contribute to slowing the translation of scientific evidence [10].

Few studies have examined optimal methods for displaying the results of clinical research reports. Prior work regarding primary literature has focused on displaying systematic reviews and investigating different methods of displaying results across studies, such as short summaries [11-13], tables [14-19], and harvest plots [20]. One recent study examined a novel presentation of clinical trial reports that restructured the visualization into several panels (ie, study purpose, process model and data grid for viewing results, statistical methods, and result interpretations). Using this visualization, translational researchers spent less time understanding and interpreting the clinical trials but maintained the same accuracy [21]. In general, very few of those studies have used any theory to drive their work.

The purpose of this study was to investigate alternative display approaches to present relevant information from randomized controlled trials (RCTs) to support clinical decision-making. Overall, we hypothesized that interactive visual displays would reduce clinicians’ cognitive workload in interpreting RCTs compared with narrative RCT abstracts. Building on Slager et al’s exploratory study on static tabular displays [22], we employed *information foraging* theory [23] and information visualization techniques to design a high-fidelity prototype with interactive visual displays of RCT results. The information displays were designed to help clinicians rapidly review, synthesize, and compare the results of relevant RCTs for the treatment of a specific patient. In this study, we described the RCT information displays and addressed the following three research questions: (1) Is the interface usable? (2) Is there a difference in *perceived efficiency*, *effort*, *effectiveness, user experience,* and *preference* between interactive visual displays and narrative abstracts? and (3) Do clinicians’ *perceived user experience*, *efficacy*, *effort,* and *effectiveness * predict their *intention to use* interactive visual displays?

### Theoretical Framework

The design of the information displays was based upon *information foraging* theory [23], Shneiderman’s information visualization principles [24], and the Population, Intervention, Comparison, and Outcome (PICO) framework [25]. Information foraging theory was initially proposed for Web designers [26]. Based on an analogy with animals’ foraging, *information foraging* theory indicates that information seekers use *information scent* (ie, cues indicating the existence of easily accessible and relevant information) to select *information patches* to explore maximizing the value (ie, *perceived utility of the information*) to cost (ie, time and effort required to explore the patch) ratio [23]. Within a certain patch, the concentration of relevant information can be increased through a process called *information patch enrichment* (eg, use of filters). Information foraging theory is grounded on the Holling Disc Equation, which equals the ratio of the total net amount of valuable information gained to the sum of the total amount of time spent between-patches and within-patches [27]. Shneiderman proposed an information visualization principle according to which information displays should first provide an information *overview,* with the ability to *zoom and filter,* and then retrieve *details on demand* [24].

RCTs are the highest-level evidence in evidence-based medicine [28]. The PICO elements are key components of the Consolidated Standards of Reporting Trials (CONSORT) statement for reporting the quality of RCTs [29]. In addition, the PICO elements have been identified in multiple studies as the critical elements of an RCT [30-32] and are almost ubiquitous within medical journal abstracts [31]. PICO has been reported to be a more effective search input format than the standard PubMed search interface for answering clinical questions [25,33]. More recently, Slager et al found that clinicians favored a PICO-based tabular display over the typical narrative abstracts reported in scientific journals [22].

## Methods

### Overview

**Figure 1:**
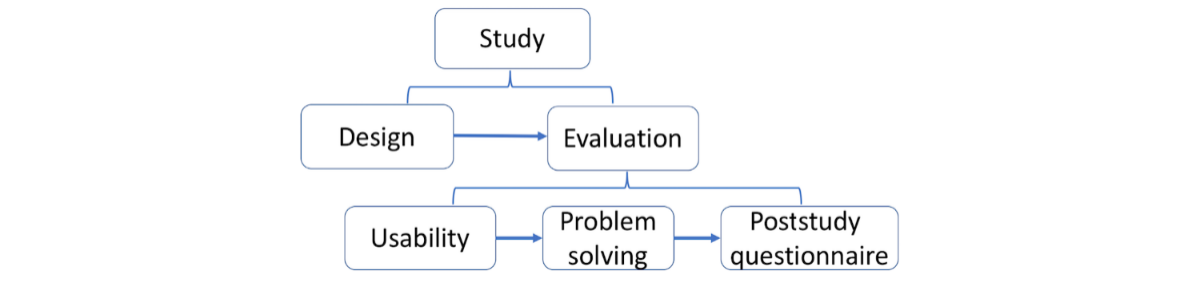
Visualization of study structure.

Our study had two phases. The first phase was the process of designing and implementing the information displays. The second was an experimental formative evaluation assessing *usability* and *problem-solving* impact. The second phase included three stages: (1) *usability* test of the interactive visual displays; (2) *problem solving* for two case vignettes comparing narrative abstracts versus interactive visual displays; and (3) a poststudy questionnaire (Figure 1).

### Phase One: Design of the Interactive Visual Displays

#### Design

We followed a user-centered and iterative design process with feedback from informatics students, clinicians, and human factor experts. The study authors (three informatics and human factors specialists and four informatics students; five with clinical backgrounds) designed the first several iterations, followed by feedback from three independent informatics researchers with clinical backgrounds.

We started with *low-fidelity* prototypes that were designed with user interface mockup software (*NinjaMock*) [34] and a free website builder (Wix) [35]. After approximately 50 design-feedback iterations, the information displays evolved into a *high-fidelity* interactive prototype: a Web-based application implemented in HTML, CSS, JavaScript, and an open-source third party graphic library called *Highcharts* [36].

#### Data Structure

First, we searched a set of RCTs in PubMed related to three clinical case vignettes that were used in the evaluation phase. Next, we manually extracted data, including PICO elements, from each of the selected RCTs. The following data were extracted from each RCT on a spreadsheet: *PMID*, *journal*, *publication date*, *study title*, *study acronym*, *population inclusion criteria*, *population age*, *study sample size*, *study country*, *aim*, and *main conclusion*. For *study arms*, we extracted the study *arm intervention* and *number of participants*. For each *study arm*, we extracted *name* and *results* of all *major outcome measures*, *overall adverse event rate,* and *most common adverse events*. Last, we designed an Extensible Markup Language (XML) schema to represent the RCT data, created XML instances for each RCT, and transformed the XML instances into Java Script Object Notation format for consumption by the application. The RCTs used in the prototype were manually searched and selected, as the purpose of the study was to compare the information displays as opposed to search engines. RCT data were manually extracted since the purpose of the study was not to investigate automated methods to extract PICO elements.

### Phase Two: Formative Evaluation Comparing Narrative Abstracts Versus Interactive Visual Displays

For formative evaluation, we used a within-subjects experimental design. The within-subjects design has advantages over a randomized, between-subjects design: it has higher statistical power, requiring a smaller sample size; and it enables participants to directly compare two designs. We tested the *usability* of the interactive visual displays and compared subjects' perceptions about narrative abstract displays versus interactive visual displays for clinical *problem solving* using case vignettes.

#### Study Setting

Formative evaluation sessions were conducted via online meetings using a Web meeting software (WebEx). Participants accessed an instance of the interactive visual displays hosted at the University of Utah Center for High Performance Computing [37]. The Uniform Resource Locator (URL) was shared with participants at the beginning of the evaluation sessions.

#### Participant Recruitment

We recruited 20 participants (8 attending physicians and 12 residents) who had not previously been exposed to the interactive visual displays (see Multimedia Appendix 1, Table A1). Participants were recruited from the Departments of Family Medicine at the University of Utah and Partners Healthcare via announcements that were sent to departmental email lists. We also employed the snowball sampling technique which asked study participants to promote the study among their colleagues. All participants received a US $100 incentive to participate in the study sessions. A previous study with a similar design [22] demonstrated that 20 participants were enough to detect a moderate difference between interactive visual displays and narrative displays with a power of 0.80.

#### Information Displays Evaluation Procedure

We randomized participants to the order of presentation of the two tools and to the vignette-tool assignment. Each session began with a brief introduction about the study and a short one-page PDF tutorial explaining how to use the interactive visual displays. To ensure consistency, verbal instructions were read from a predefined script for each session. Each formative evaluation session included three stages: (1) *usability* test of the interactive visual displays; (2) *problem solving* for two case vignettes comparing narrative abstracts versus interactive visual displays; and (3) a poststudy questionnaire assessing the participant’s perception of the *efficiency*, *effort*, *effectiveness*, *user experience,* and *preference* of interactive visual displays versus narrative abstracts. In the first two stages, the participants were asked to share their screens via WebEx, and their screen interactions were recorded for data analysis. In the third stage, participants were asked to stop screen-sharing while answering the poststudy questionnaire to ensure anonymity and minimize the Hawthorne effect. A waiver of written consent was approved by the Institutional Review Board of the University of Utah. Participants provided verbal consent before the study session.

##### Case Vignettes and RCTs

We prepared three case vignettes (Table 1), which presented challenges related to patient treatment. The vignettes were obtained from the literature and adapted by clinicians in our team. For each case vignette, we searched for potentially relevant RCTs using PubMed’s Clinical Query treatment filter and a Medical Subject Heading (MeSH) term that matched the main disease of the case.

**Table 1:** Case vignettes used in the formative evaluation.

Case vignette	Stage Used	Complexity^a^	Designer	Number of studies
Acute coronary syndrome	*Usability*	Easy	Article author (PU^b^)	2
Rheumatoid arthritis	*Problem solving*	Complex	Adapted from Medscape [38]	10
Diabetes mellitus	*Problem solving*	Complex	Adapted from Hirsch et al [39]	10

^a^The complexity level of each vignette was determined by the number of factors involved in each treatment case.

^b^PU refers to co-author Prasad Unni.

We manually screened the retrieved articles for RCTs on the diseases of interest and presented the same RCTs in the same order, both within PubMed and the interactive visual displays. The case vignettes and selected RCTs are available in Multimedia Appendix 1 (case vignettes).

##### Stage One: Usability of Interactive Visual Displays

We developed 19 tasks to test the *usability* of the key information and features provided by interactive visual displays. Most of the tasks required participants to perform an action (eg, *highlight*, *access, switch*). We read the tasks aloud one-by-one to each participant and let them complete the tasks independently without assistance. We measured the *time spent* and *success* on each task. We also tape recorded the session and transcribed participants’ comments.

##### Stage Two: Problem Solving

Participants were told that the rheumatoid arthritis and diabetes mellitus cases are relatively complex and that there are multiple reasonable treatment options for each case. We asked each participant to complete this stage in no more than 10 minutes in order to simulate the time pressure of a real patient visit [1,40]. We notified participants when there were 3 minutes and 1 minute left to finish the session. Within each case vignette session, participants could go back to the vignette description at any time.

For PubMed, participants were given a hyperlink that gave access to a *search results* page with the 10 RCTs in PubMed’s default search results display format (see Multimedia Appendix 1, Figure A1). No washout time was provided between the two case vignettes. At the end of the *problem-solving* stage, we asked each participant to provide a summary of the evidence they found and their decision about the treatment for the patient.

##### Stage Three: Participant Information-Seeking Experience Questionnaire

In this stage, we asked participants to complete an online REDCap [41] questionnaire regarding their information-seeking experiences with the tools. The questions (see Multimedia Appendix 1, “Post Evaluation Survey”) were adapted from the System Usability Scale [42], the National Aeronautics and Space Administration (NASA) Task Load Index (NASA-TLX) tool [43], and from Slager et al [22]. Two versions of the questionnaire were used, depending on which tool the participant was randomly assigned to use first. Each participant only needed to complete one survey.

The questionnaire started with items about participants’ demographics, *experience with cases in the domain of the vignettes,* and *experience with literature searching*. Next, using 17 Likert scale items, participants were asked to rate interactive visual displays versus narrative abstracts according to *perceived efficiency*, *effectiveness*, *effort*, *user experience*, and *preference*. The anchors for each question juxtaposed narrative abstracts in PubMed on one end and interactive visual displays on the other with the direction of the anchor randomized. For example, the hypothesis that there would be a difference in *perceived effectiveness* for users between interactive visual displays and narrative abstracts was assessed by four survey items: (1) *comprehend the meaning of the information presented well*, (2) *identify relevant information to understand the study*, (3) *effectively identify relevant RCTs from the search results*, and (4) *accomplish tasks with minimal frustration*. The results were constructed so that there were separate ratings for narrative abstracts and interactive visual displays for each question by centering the scores for both displays separately. Participants were then asked to rate the *intention to use* and *learnability* of using interactive visual displays on a 1 (“Strongly Disagree”) to 9 (“Strongly Agree”) scale. Scales were created for each of the constructs as the sum of the ratings given to each of the items in the construct. We reported internal reliability for the scales using Cronbach alpha (Table 2). Last, participants were asked to provide suggestions for improving interactive visual displays.

### Data Analysis

We analyzed the *usability* results and the Likert scale items in *problem solving* to address the following questions below. We performed all statistical analyses using IBM SPSS Statistics Premium 24 [44].

#### Is the Interface Usable?

We conducted both qualitative and quantitative analyses to answer this question. We employed a qualitative analysis software (ATLAS.ti [45]) to code, categorize, and analyze users’ verbalizations in the usability stage. To establish reliability for success and time measures, two authors (JB and DB) developed and tested coding protocols and employed the Cohen kappa and Pearson’s correlation coefficient (PCC) to measure the interrater agreements. When both measurement metrics reached 0.80, we split the remaining sessions between coders in order to reduce the workload. For each task, we reported the mean, standard deviation, median, and range for the time spent and reported the success rate. We analyzed the correlation between the *experience with literature searching* and success rate of the usability tasks, and also categorized the open-ended comments and reported the descriptive statistics.

**Table 2:** Construct items and Cronbach alpha.

Construct and items		Cronbach alpha
**Experience with cases in the domain of the vignettes**		.793
	Dealing with patients in the same clinical domain of the narrative abstracts case vignette	
	Dealing with cases with similar clinical complexity as in the case presented in the narrative abstracts vignette	
	Dealing with patients in the clinical domain of the interactive visual display case vignette	
	Dealing with cases with similar clinical complexity as in the case presented in the interactive visual display vignette	
**Experience with literature searching**		.870
	Experience in using computers for work activities	
	Experience in using medical literature search tools in general (eg, PubMed, UpToDate)	
	Experience in using PubMed for medical literature search	
**Efficiency**		.877
	Scan the information quickly	
	Quickly obtain the gist of the study findings	
	Locate information rapidly	
	Interpret individual RCT^a^ results quickly	
	Quickly compare the results of multiple RCTs	
	Quickly determine study relevance for the case vignette	
**Effectiveness**		.921
	Comprehend the meaning of the information presented well	
	Identify relevant information to understand the study	
	Effectively identify relevant RCTs from the search results	
	Accomplish tasks with minimal frustration	
**Effort**		.823
	Spend the least degree of mental effort	
	Accomplish task effortlessly	
**User experience**		.921
	Be satisfied with the presentation (ie, format of the display) of the information	
	Easily use the user interface	
	Enjoy exploring information	
	Have fun seeking information to find answers	
**Intention to use**		.971
	Help me with clinical decisions for specific patients	
	Find evidence during patient consultations	
	Find evidence after patient consultations	
	Prepare for patient appointments	
	Prepare for patient rounds	
	Prepare for teaching	

^a^RCT: randomized controlled trial.

#### Is There a Difference in Perceived Efficiency, Effort, Effectiveness, User Experience and Preference Between Interactive Visual Displays and Narrative Abstracts?

We employed the paired *t*-test to assess differences in ratings for each variable. We also assessed if there was a difference between interactive visual displays and narrative abstracts after controlling for years of expertise, tool presentation order, clinical role, experience with literature searching, and experience with cases in the domain of the vignettes.

#### Do Clinicians' Perceived User Experience, Efficacy, Effort, and Effectiveness Predict Their Intention to Use Interactive Visual Displays?

To answer this question, we regressed intention to use on user experience, efficacy, effort, and effectiveness.

## Results

### Interactive Visual Displays

**Figure 2:**
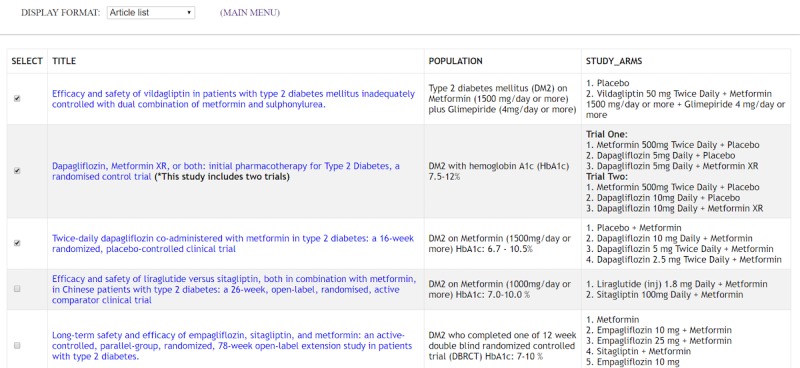
*Article list* table with trials on various treatments for diabetes mellitus. This display is the landing page of the information displays. It provides a table with *the title*, patient *population*, and *study arms* of each study. The goal is to allow clinicians to quickly scan each study and select relevant ones for further review.

**Figure 3:**
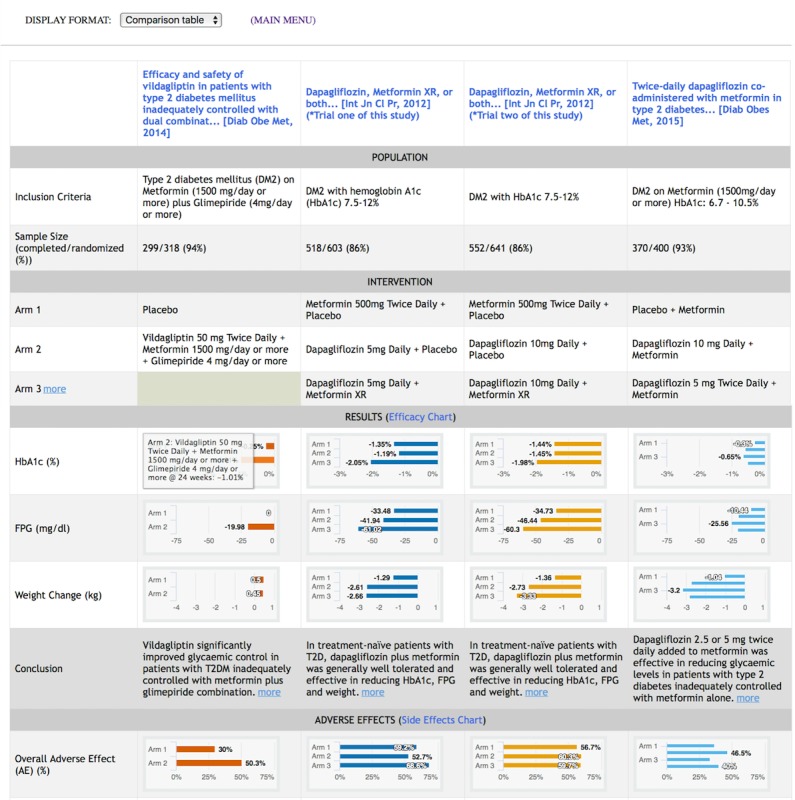
*Comparison table* display with four trials on various treatments for diabetes mellitus. This display contains key elements of selected studies in a tabular format according to the Population, Intervention, Comparison, Outcomes (PICO) framework [29-32]. Studies are displayed in columns, and attributes of studies are displayed in rows. Study results for primary outcomes and adverse events are represented in bar graphs [46]. Hovering over a bar brings up a callout with details on the intervention of the selected study arm. The scale of each measure is normalized across all studies to enable direct visual comparison. An illustration of the *comparison table* display for randomized controlled trials on rheumatoid arthritis is available in Multimedia Appendix 1, Figure A2.

**Figure 4:**
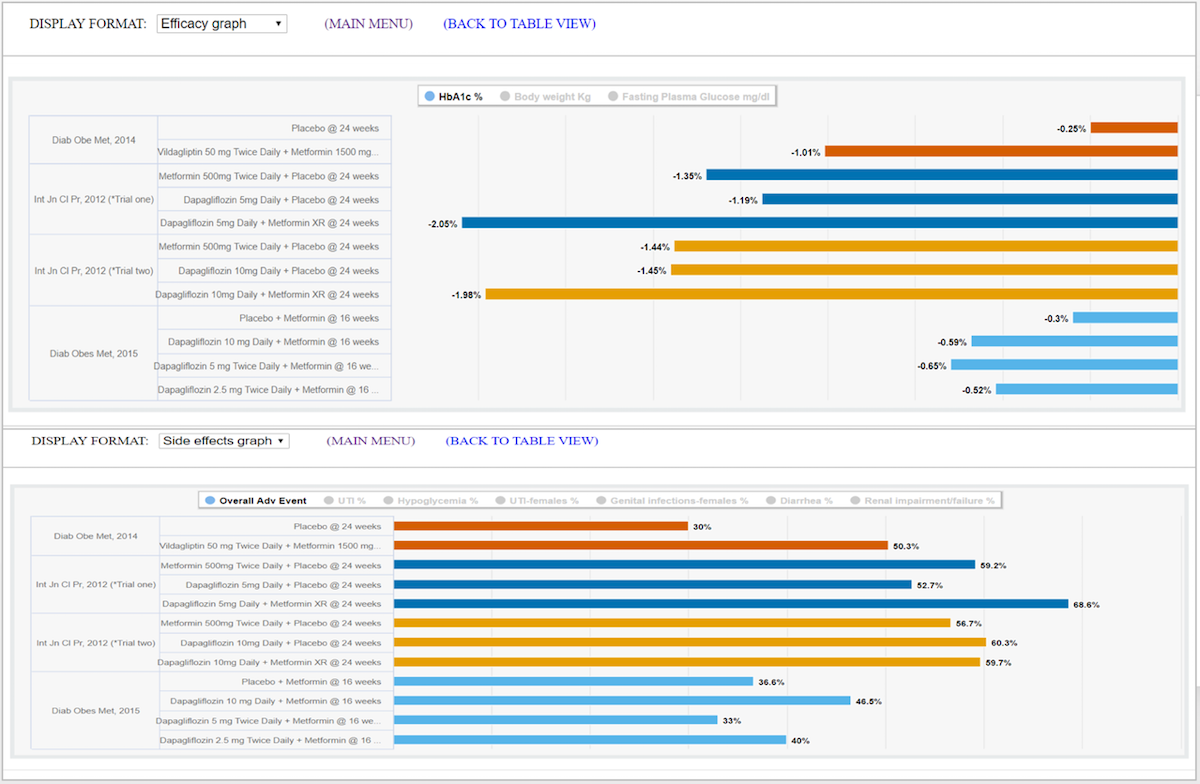
*Efficacy graph* display (top) and *side effects graph* display (bottom) with four trials on various treatments for diabetes mellitus. These two displays provide graphical comparisons of study primary outcomes and adverse effects respectively. Users can choose to set the bar graph for a specific outcome measure, overall adverse effects, or the most common adverse effect across all the arms of the selected studies.

Following Shneiderman's principles, the interactive visual displays provided information *overviews* and *filters* with the option to retrieve *details on-demand*. These principles guided the design of each of the features in Table 3. These features are operationalized in one of five information displays, which are *article list*, *text summary*, *comparison table*, *efficacy graph,* and *side effects graph*. The displays can be launched for each case vignette by clicking on the “i” icons at our website [37]. Figures 2-4 depict the features listed in Table 3. A drop-down menu is used for switching between five information displays. *Article list* and *text summary* information displays aim to help users judge the relevance of an RCT based on the study patient characteristics and the interventions under investigation. The *comparison table, efficacy graph,* and *side effects graph* information displays allow users to compare the results of relevant RCTs side-by-side. To avoid visual cluttering, we limited the maximum number of studies to *four* that can be displayed in the *comparison table*, *efficacy graph,* and *side effects graph* information displays.

### Formative Evaluation

The formative evaluation results are structured according to the research questions.

#### The Interface is Usable

After two WebEx recording sessions (one resident and one attending) were coded and analyzed by DB and JB independently, the interrater agreement Cohen kappa (1.00) and PCC (0.92) were higher than the threshold established *a priori*, so we split the coding of the remaining recording sessions between two coders. Overall, the participants were able to solve each of the 19 *usability* tasks within a *median time* of 3 to 11 seconds, and a *success rate* of 78% to 100% (Table 4).

**Table 3:** Design principles that inspired each feature in the interactive visual displays.

Information display	Feature	Design principle
*Article list*	Information about study population and interventions	*Information scent*
*Article list*	Hyperlink to full abstract within PubMed	*Details on demand*
*Article list*	Ability to select specific, most relevant studies for further visualization	*Information patch enrichment, filter* and *zoom*
*Comparison table*	Population, Intervention, Comparison, and Outcome (PICO) table structure	*Information scent*
*Comparison table*	Hyperlink to full abstract within PubMed and hyperlink to efficacy and side effect graph	*Details on demand* and *zoom*
*Efficacy graph/side effects graph*	Ability to choose different outcome measures or side effects	*Information scent* and *zoom*

*Experience with literature searching* was modestly correlated with *success* for the *usability* tasks (*r*=0.417, *P*=.10). A total of 14 out of 20 participants responded to the *open-ended comments* section, which we analyzed into categories. “*Great tool”* was the most frequent comment category (6 out of 14 participants), followed by “*allow more than 4 studies for comparison”* (5 out of 14 participants). Other less frequent comment categories included: *request for more features*, *request for more information*, *request for clearer display*, and *prefer narrative abstracts in PubMed* (see Multimedia Appendix 1, Table A2).

#### Clinicians Favored Interactive Visual Displays Over Narrative Abstracts on Perceived Efficiency, Effectiveness, Effort, User Experience, and Preference

**Figure 5:**
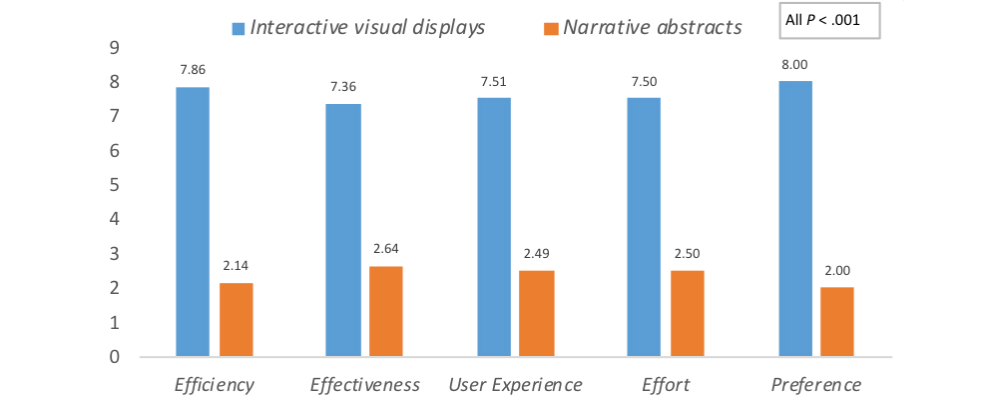
Mean differences for participants’ *perceived efficiency, effectiveness, effort, user experience* and *preference* of interactive visual displays versus narrative abstracts.

The paired *t*-test results showed that clinicians favored interactive visual displays over narrative abstracts on all of the variables: *efficiency* t_(18)_=10.43 (mean 7.86 vs 2.14, respectively), *effectiveness* t_(19)_=6.90 (mean 7.36 vs 2.64), *effort* t_(19)_=8.24 (mean 7.50 vs 2.50), *user experience* t_(19)_=7.94 (mean 7.51 vs 2.49), and *preference* t_(19)_=8.62 (mean 8.00 vs 2.00). All differences were significant (*P*<.001). Figure 5 displays the comparison results.

In addition, participants’ *years of expertise, tool presentation order, clinical role, experience with literature searching,* and *experience with cases in the domain of the vignettes* were not correlated with any of the participants’ perception variables (all *P* values >.05; Multimedia Appendix 1, Table A3), which indicates that there is no need to control for these factors when comparing the difference between the two tools.

#### Do Clinicians' Perceived User Experience, Efficacy, Effort, and Effectiveness Predict Their Intention to Use Interactive Visual Displays?

A scale for *intention to use* was created from six variables and had a Cronbach alpha of .971 (Table 2). We regressed *intention to use* on *user experience*, *efficacy*, *effort,* and *effectiveness.* The stepwise linear regression, which removes the variable with the highest beta weight sequentially, showed that *efficiency* was the only item that entered the prediction model (R^2^_(16)_=0.661, *t*=5.59, F_(16)_=31.26 and *P*<.001) after controlling for all others.

**Table 4:** Time to completion (in seconds) and completion success rate for 19 usability tasks.

Usability task		Average time, seconds (SD)	Median time, seconds (range)	Success rate (%)
**On the *Article list* format**				
	Highlight the study arms of the first study	7 (5)	5 (3-18)	83
	Highlight the population of the second study	7 (14)	3 (1-59)	89
	Access the PubMed abstract of the first study	7 (6)	4 (3-21)	82
	This tool provides a textual summary of RCTs^a^. Please find out how to switch to the text summary of the two listed studies.	4 (1)	4 (2-6)	83
**On the *Text summary* format**				
	What is the RCT publication journal and year of the first study?	7 (6)	5 (2-26)	100
	Highlight the aim and conclusion of the second study	3 (1)	4 (1-6)	100
	This tool also provides comparison views for multiple RCTs. Please switch to the comparison view for the two listed studies.	4 (3)	3 (1-13)	94
**On the *Comparison table* format**				
	Highlight the study population of the first study	6 (2)	6 (2-10)	83
	Highlight the study with the largest sample size	4 (3)	4 (1-14)	100
	Highlight the research arms of the second study	5 (2)	5 (2-9)	94
	Identify one of the study endpoints reported in both studies	8 (5)	7 (3-20)	83
	Within trial 1, which *drug therapy* resulted in greatest total cholesterol reduction?	11 (10)	9 (3-44)	78
	Which *drug therapy* across the two trials showed the best response in terms of total cholesterol reduction?	6 (4)	5 (2-17)	78
	Which *drug therapy* across the two trials showed the best response in terms of high density lipoprotein increase?	12 (7)	11 (4-25)	100
	Highlight the conclusion of the first trial	4 (5)	3 (1-23)	100
	This tool also provides graphical visualization of RCTs. Please switch to the graphical view.	5 (3)	5 (2-12)	89
**On the *Efficacy graph* format**				
	Set the graph to show LDL^b^ outcomes	4 (2)	3 (1-8)	83
	Which drug regimen across the two trials showed the greatest reduction in LDL?	4 (2)	3 (1-10)	100
	Switch back to the main menu	3 (1)	3 (1-6)	100

^a^RCT: randomized controlled trial.

^b^LDL: low density lipoprotein.

## Discussion

### Summary

The goal of this work was to design a novel information display to help clinicians interpret, compare, and apply evidence from RCTs in clinical settings. We previously investigated a static structured PICO table for representing clinical trial reports and found that clinicians preferred a tabular PICO display over PubMed’s default search results display [22]. We choose PubMed’s default search results display as a baseline based on two reasons: (1) PubMed is the most widely used resource for browsing the biomedical literature, including RCT publications; and (2) PubMed is representative of resources in the same category (eg, Ovid, EBSCO, Scopus) since biomedical literature databases rely on the same narrative abstracts provided by biomedical journals. In this current study, we added graphical and interactive features to the previous static structured display and conducted a formative evaluation with 20 physicians in a simulation setting with case vignettes. Our results showed that when interpreting and applying research findings to patient care, physicians strongly preferred interactive visual displays that enable direct comparison of the results from multiple RCTs over narrative abstracts.

### Information Processing Issues

Our findings suggest that the cognitive tasks involved in reviewing the literature are perhaps more complex than we had previously been aware. The tasks may involve a compilation of information processing goals (epistemic goals) that vary according to the clinical situation. Human information processing is essentially goal-oriented, so tailoring information to address specific goals is important [47]. Prior work in this area has found that task *problem-solving* is the most common information need in this context [48]. Our work suggests that displays that show adverse events, results by specific outcomes, and population descriptions by experimental arm match the information processing goals of clinicians seeking research information for medication decision-making.

In addition to exploring information processing goals, our results also suggest the need for further exploration of risky decision-making in work settings. One area that might be particularly fruitful is the well-established and robust findings from research in the “description-experience gap” [49]. This body of research has found large differences in decision-making between choices based on experience versus choices based on the provision of descriptive information. In general, physicians may weigh the probability of a loss (adverse events) and gain (treatment effectiveness) differently when being presented evidence rather than from their experience. Examining how displays can improve the accuracy of decision-making probability estimates is also an area of further research [49].

Information Foraging and information visualization principles (listed in Table 3) guided this study. Participants’ preference for interactive visual displays can be attributed to the following reasons. First, interactive displays, as the central piece of visual analytics [50], provide clinicians with multiple advantages [51,52]. For example, with the interactive functions, only relevant information is presented up-front, and further details can be provided on demand. Second, the use of graphics reduces clinicians’ cognitive effort when interpreting the results of multiple clinical trials [53]. In our displays, users can make direct comparisons both between and within clinical trials on the same display, thereby minimizing working memory overload [54]. Third, the PICO framework has been recommended to clinicians when formulating evidence-based clinical questions [25]. Therefore, our PICO tabular displays provide a consistent structure that is compatible with clinicians’ mental models, facilitating their understanding of the gist of the evidence presented in RCTs [55].

### Technology Adoption

According to the technology acceptance model proposed by Davis [56] and expanded on by the unified theory of acceptance and use of technology [57], *perceived ease-of-use* (PEOU), performance beliefs (ie, how well does it help me do my task), *perceived effort*, and social norms predict the actual use of a new technology. Findings from our *usability* study (PEOU) suggest that the prototype is easy to use. Most participants completed the *usability* task correctly within a short period of time, with minimal training. In the clinical *problem-solving* session (performance beliefs and effort), participants’ preference of the interactive visual displays was significantly higher than the narrative displays according to several perceived ability measurements. The performance of perceived ability measurements was not correlated with any of the clinicians’ characteristics, suggesting that our finding is generalizable to a different range of users. The within-subjects design with randomized vignette assignment and tool presentation order minimized the impact of the participant’s individual differences. We did not measure social norms. In sum, the interactive visual displays have the potential to ease clinicians' effort to interpret evidence from the primary literature at the point of care.

The stepwise regression analysis of the clinical *problem-solving* stage showed that *efficiency* was the only factor that predicted *intention to use.* Multicollinearity analysis also showed that only one dimension exists, which means that all predicting factors are correlated with each other. It is likely that *perceived efficiency* or *effectiveness* is the most general latent variable. It is possible that all of the poststudy questions measure the participants’ general attitude towards the tool with little distinction among factors.

### Implications for the Reporting of RCTs

Our study findings add to the growing evidence supporting alternative information display formats to convey the gist of clinical studies [11-22], suggesting that the standard format of scientific reporting, especially for article abstracts, is worth reconsidering. The ideal abstract display format should match clinicians’ mental models to reduce cognitive workload in interpreting clinical study results. Much progress has been made with the increased adoption of structured abstracts, which are more readable, easier to search, preferred by readers, and easier to peer review than traditional unstructured abstracts [58,59]. Our findings suggest that interactive visual displays could further improve the presentation of summaries of clinical studies.

One important challenge in enabling interactive visual displays of clinical studies is the lack of a widely adopted standard data model for reporting study methods and results in a computable format. National clinical trial registries such as ClinicalTrials.gov have taken an important step towards the implementation of structured reporting. However, several challenges still exist, such as automatically extracting key study data from clinical trial registries [60], incomplete linkage between clinical trial publications and clinical trial registration [61], and time delay between clinical trial publication and reporting of results in clinical trial registries [60]*.* Increasing requirements for structured reporting of clinical trials could be a possible solution. For example, core clinical journals could adopt and require structured reporting of clinical trial results using a common computable data model.

### Limitations

This study has several limitations. First, we have not analyzed how much time participants spent looking at each component or piece of information in the information displays. Methods based on eye-tracking devices can be used in future studies to provide deeper insight into how users process the information presented on the screen. Second, the case vignettes did not have a single right or wrong answer, so it was impossible to measure the effect of the interactive visual displays on the accuracy of clinical decisions. Nevertheless, the vignettes were purposefully complex to stimulate a challenging information-seeking experience. Third, in this simulation study, we limited the information displays to 10 studies per case vignette. In real search sessions, the number of studies in a search result can be much higher.

### Future Work

The RCT data under the interactive visual displays were manually extracted from a limited set of hand-selected RCTs. Future work is needed to automate the RCT data extraction process, leveraging resources such as ClinicalTrials.gov or RCT data extraction algorithms [60,62-64]. This work is underway, with a prototype currently available. Future studies should also implement the interactive visual displays in clinical settings and investigate their effect on clinicians’ patient care decisions and clinical outcomes.

### Conclusion

This study shows that when interpreting and applying research findings to patient care, physicians preferred graphical, interactive, and PICO-framework-based information displays that enable direct comparison of the results from multiple RCTs compared to the traditional narrative format of article abstracts. Future studies should investigate the use of these displays in clinical care settings and their effect on improving clinicians’ patient care decisions and clinical outcomes.

## Appendix

Multimedia Appendix 1 Supplementary tables and figures, case vignettes, and post evaluation survey.

## Abbreviations

CONSORT: Consolidated Standards of Reporting Trials
LDL: low density lipoprotein
MeSH: medical subject heading
NASA: National Aeronautics and Space Administration
PCC: Pearson’s correlation coefficient
PEOU: perceived ease-of-use
PICO: Population, Intervention, Comparison, and Outcome
RCT: randomized controlled trial
URL: Uniform Resource Locator
XML: Extensible Markup Language
