# Supplementary material for: Interactive Visual Displays for Interpreting the Results of Clinical Trials: Formative Evaluation With Case Vignettes
Source: J Med Internet Res. 2018 Jun 25;20(6):e10507. doi: 10.2196/10507 (PMC6037946; doi:10.2196/10507)
Supplement: Multimedia Appendix 1 [file jmir_v20i6e10507_app1.pdf]

Table A1. Evaluation participants

ID	Gender	Clinical role	Specialty	Years of practice
1	F	Resident	Family Practice	2
2	F	Resident	Family Medicine	2
3	M	Resident	Family Medicine	1
4	F	Resident	Family Medicine	3
5	F	Resident	Family Practice	2.75
6	M	Attending	Anesthesiology	5
7	F	Attending	Cardiology	10
8	M	Resident	Emergency Medicine	4
9	M	Attending	Internal medicine hospitalist	15
10	M	Attending	Internal Medicine	21
11	M	Resident	Family Medicine	2
12	F	Resident	Family Medicine	3
13	F	Resident	Family Medicine	2
14	M	Resident	Family Medicine	2.75
15	M	Attending	Internal medicine and pediatrics	13
16	M	Resident	Internal Medicine	1
17	M	Resident	Internal Medicine	1
18	M	Attending	Nephrology/Int Med	43
19	F	Attending	Internal medicine	24
20	F	Attending	Geriatrics	17

Table A2. Open-ended comments and category

Comments	Category
The legends and keys for the bar graphs are a little hard to see, it seems they require dragover with the cursor, it would be nice if they were clearly displayed so I knew what the bars meant intuitively.	Request for clearer display
Provide serial numbers in the first column. (I) will be happy to be involved in further refining this tool later as well.	Request for more information
Make sure things all show up appropriately: in the DM interactive visual display the side effect UTI-female was two tabs so different studies had their results show up under different tabs instead of showing up together and being comparable. It was a really cool comparison tool! It would be great if it were possible to compare more than 4 studies at a time.	1) Great tool; 2) Allow more than 4 studies for comparison; 3) Request for clearer display
It would be nice to have a select all or de-select all check boxes on the left-hand side.	Request for more features

It was very effective as a clinical tool. I cannot think of any improvements.	Great tool
It was great, I can imagine using this tool all the time in my practice!	Great tool
It is awesome! Maybe a way to display more RCTs at once with the comparison tools.	1) Great tool; 2) Allow more than 4 studies for comparison
Include more than 4 studies at a time	Allow more than 4 studies for comparison
I would have liked to see error bars on some of the graphs. In these vignettes, the 10 studies were provided, but how will you propose coming up with those 10 studies in real-life clinical practice?	Request for more information
I think that it would be nice to make graphical comparisons of more than 4 studies at once if need be	Allow more than 4 studies for comparison
I am very familiar with abstract format, so I think that is why I liked PubMed. I question whether the right trials were included in interactive visual display. I have control over this is PubMed search. A quick link to the right clinical guidelines may be more helpful than the actual RCTs because I know the process by which that data is vetted for accuracy and I know I would practicing in accordance with current standards.	Prefer narrative abstracts in PubMed
great tool, less incentive to read methods and figure out the validity of a study as the results are prominently displayed but could easily do. if simple validity criteria are available that would be really something.	1) Great tool; 2) Request for more information
Continue to maintain easy use of the tool.	Great tool
Allow the option to select more than 4 trials which will give a better view overall. Put select all option in the right-hand corner to quickly deselect all selected options	1) Allow more than 4 studies for comparison; 2) Request for more features

Table A3. Correlation analysis between *years of expertise*, *tool presentation order*, *clinical role*, *experience with literature search*, *experience with cases in the domain of the vignettes* and perception variables

		Years of expertise	*Tool presentation order*	*Clinical role*	*Experience with cases in the domain of the vignettes*	*Experience with literature search*
Efficiency	Pearson Correlation	0.124	-0.189	-0.218	0.011	-0.112
	Sig. (2-tailed)	0.623	0.439	0.371	0.965	0.659
	N	18	19	19	18	18
Effectiveness	Pearson Correlation	0.212	-0.191	-0.079	-0.007	-0.195
	Sig. (2-tailed)	0.384	0.42	0.742	0.977	0.423
	N	19	20	20	19	19
Effort	Pearson Correlation	0.086	-0.254	-0.193	-0.041	-0.116
	Sig. (2-tailed)	0.728	0.281	0.415	0.869	0.637
	N	19	20	20	19	19
User Experience	Pearson Correlation	0.359	-0.125	-0.028	0.055	-0.167
	Sig. (2-tailed)	0.144	0.611	0.909	0.829	0.507
	N	18	19	19	18	18
Preference	Pearson Correlation	0.166	-0.177	-0.269	-0.016	-0.143
	Sig. (2-tailed)	0.497	0.456	0.251	0.947	0.559
	N	19	20	20	19	19

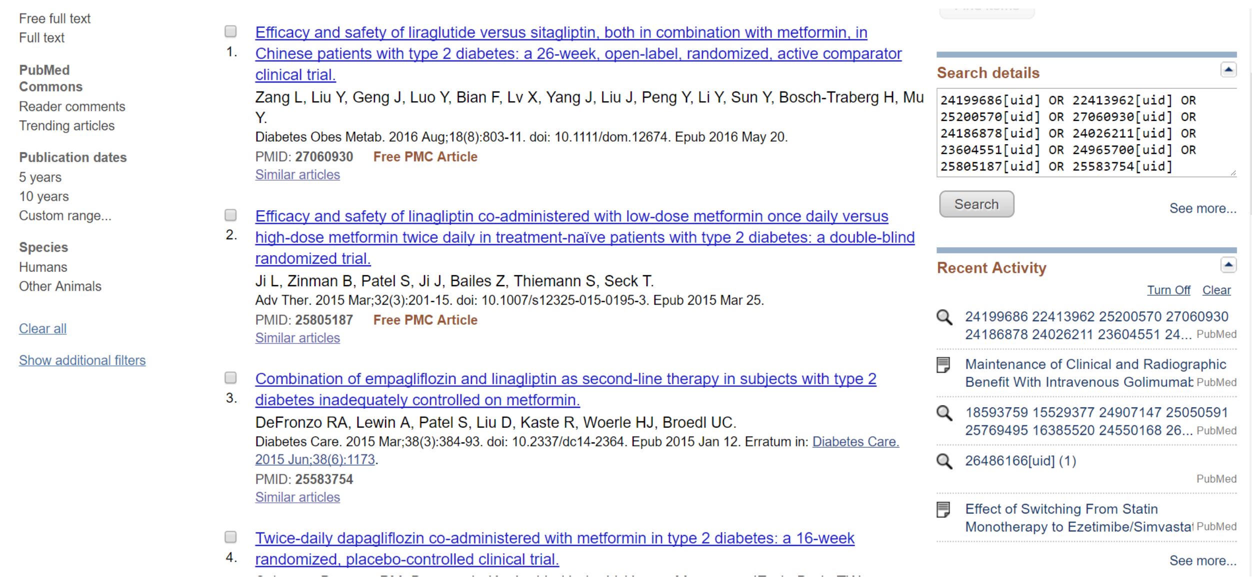

Figure A1. PubMed® default search results display with four trials on various treatments for diabetes mellitus (https://www.ncbi.nlm.nih.gov/pubmed/?term=25200570+27060930+25805187+25583754%5Buid%5D)

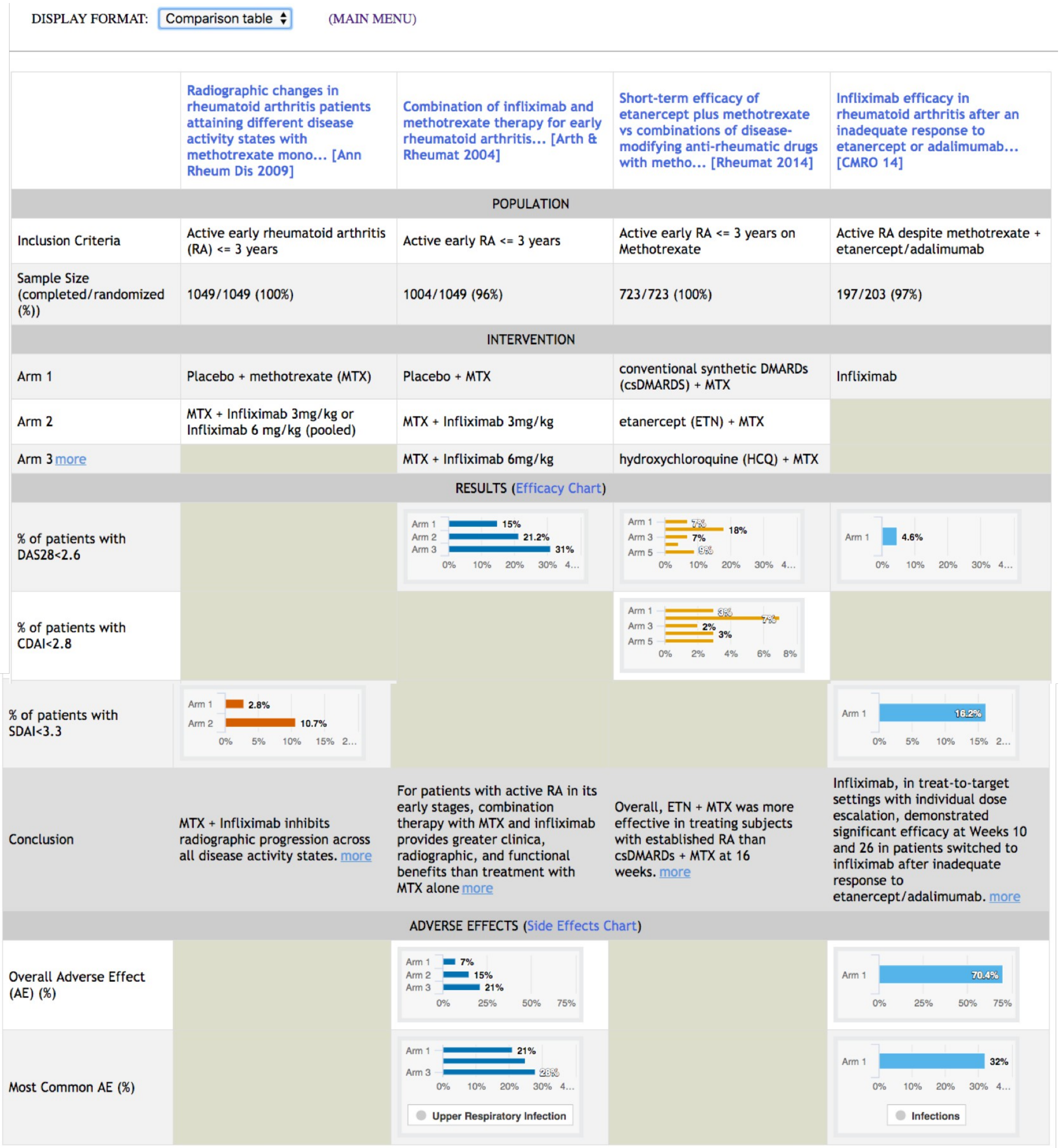
DISPLAY FORMAT: Comparison table (MAIN MENU)

	Radiographic changes in rheumatoid arthritis patients attaining different disease activity states with methotrexate mono... [Ann Rheum Dis 2009]	Combination of infliximab and methotrexate therapy for early rheumatoid arthritis... [Arth & Rheumat 2004]	Short-term efficacy of etanercept plus methotrexate vs combinations of disease-modifying anti-rheumatic drugs with metho... [Rheumat 2014]	Infliximab efficacy in rheumatoid arthritis after an inadequate response to etanercept or adalimumab... [CMRO 14]
POPULATION				
Inclusion Criteria	Active early rheumatoid arthritis (RA) <= 3 years	Active early RA <= 3 years	Active early RA <= 3 years on Methotrexate	Active RA despite methotrexate + etanercept/adalimumab
Sample Size (completed/randomized (%))	1049/1049 (100%)	1004/1049 (96%)	723/723 (100%)	197/203 (97%)
INTERVENTION				
Arm 1	Placebo + methotrexate (MTX)	Placebo + MTX	conventional synthetic DMARDs (csDMARDS) + MTX	Infliximab
Arm 2	MTX + Infliximab 3mg/kg or Infliximab 6 mg/kg (pooled)	MTX + Infliximab 3mg/kg	etanercept (ETN) + MTX	
Arm 3 more		MTX + Infliximab 6mg/kg	hydroxychloroquine (HCQ) + MTX	
RESULTS (Efficacy Chart)				
% of patients with DAS28<2.6		Arm 1 15%; Arm 2 21.2%; Arm 3 31% (axis 0% 10% 20% 30% 4...)	Arm 1 7%; 18%; Arm 3 7%; Arm 5 9% (axis 0% 10% 20% 30% 4...)	Arm 1 4.6% (axis 0% 10% 20% 30% 4...)
% of patients with CDAI<2.8			Arm 1 3%; 7%; Arm 3 2%; 3%; Arm 5 (axis 0% 2% 4% 6% 8%)	
% of patients with SDAI<3.3	Arm 1 2.8%; Arm 2 10.7% (axis 0% 5% 10% 15% 2...)			Arm 1 16.2% (axis 0% 5% 10% 15% 2...)
Conclusion	MTX + Infliximab inhibits radiographic progression across all disease activity states. more	For patients with active RA in its early stages, combination therapy with MTX and infliximab provides greater clinica, radiographic, and functional benefits than treatment with MTX alone more	Overall, ETN + MTX was more effective in treating subjects with established RA than csDMARDs + MTX at 16 weeks. more	Infliximab, in treat-to-target settings with individual dose escalation, demonstrated significant efficacy at Weeks 10 and 26 in patients switched to infliximab after inadequate response to etanercept/adalimumab. more
ADVERSE EFFECTS (Side Effects Chart)				
Overall Adverse Effect (AE) (%)		Arm 1 7%; Arm 2 15%; Arm 3 21% (axis 0% 25% 50% 75%)		Arm 1 70.4% (axis 0% 25% 50% 75%)
Most Common AE (%)		Arm 1 21%; Arm 3 28% (axis 0% 10% 20% 30% 4...); Upper Respiratory Infection		Arm 1 32% (axis 0% 10% 20% 30% 4...); Infections

Figure A2. *Comparison table* display for rheumatoid arthritis RCTs

<u>Case Vignettes</u>

Acute Coronary Syndrome

A 56-year-old Chinese patient, Mr. Lee presents to the Emergency room with symptoms of acute coronary syndrome. He is seen by a resident who notices an elevated lipid profile. He says that he is on some lipid lowering therapy but more details are not available. The resident wants to find out the best lipid lowering therapy that he could start Mr. Lee on, in this situation.

Which lipid lowering drug should the resident choose?

***Clinical Trial Studies PMID*:** 26486166 and 25879728.

Rheumatoid Arthritis

Mary is a 34-year-old Caucasian woman diagnosed with rheumatoid arthritis (RA). Her initial treatment was methotrexate (MTX) 15 mg/wk orally for 4 weeks, with escalation to 20 mg/wk for another 4 weeks, and then a maintenance dose of subcutaneous MTX 25 mg/wk.

At the 6-month follow-up visit, she reports an improved overall assessment of disease activity. However, she is still experiencing some morning stiffness and functional limitations. Inflammation of joints in her hands has resulted in a loss of the ability to work. Radiographic results indicated several erosions of MCPs in each hand; her ESR was 39 mm/h and C-reactive protein (CRP) was 3.3 mg/dL. She had 8 tender and 11 swollen joints, and the disease activity score (DAS28) was 5.76, indicative of high disease activity.

The rheumatologist decides to enhance the current treatment of MTX monotherapy and considers the options of adding an additional disease-modifying antirheumatic drug (DMARD). The ideal drug of choice for Mary would one that would increase her function, reduce disease activity, and reduces radiological disease progression, while minimizing the possibility of adverse reactions.

***Clinical Trial Studies PMID*:** 18593759, 15529377, 24907147, 25050591, 25769495, 16385520, 24550168, 26063454, 24670196 and 25623393.

Diabetes Mellitus

Agnes is a 51-year-old female with hypertension who received a diagnosis of type 2 diabetes a decade ago. She has been worried about her diabetes since then because she has not been able to gain

complete control over it. Her HbA1c was 7.0% for 1 year but gradually increased to 9.0%. For the past 2 years, she has been taking metformin 2000 mg daily. She is maintaining her weight at 165 pounds (75 kg), but she is not able to lose weight. Agnes goes to the gym and walks on a treadmill three times a week. She tells you that she has made as many lifestyle changes as she can. Agnes' hypertension is well controlled with an ACE inhibitor; she also takes a statin. Her most recent tests showed LDL cholesterol of 85 mg / dL and HDL of 62 mg / dL.

Agnes hates needles and won't use insulin. Her sister, who also has diabetes, was receiving glipizide but had episodes of hypoglycemia while taking that drug, including one episode that resulted in an auto accident. Agnes is also worried about weight gain associated with that drug. Her sister recently switched from glipizide to saxagliptin and has had no further episodes of hypoglycemia. Agnes has also heard about a new type of drug that works by eliminating excess glucose through the urine. She wants to know about the safety of the newer drugs. You explain to her that the drugs she is asking about are in different classes - dipeptidyl peptidase 4 (DPP-4) inhibitors ("gliptins") and sodium glucose cotransporter 2 (SGLT2) inhibitors ("gliflozins").

Do you think a second drug should be added to the metformin Agnes is currently receiving? If so, what drug?

***Clinical Trial Studies PMID*:** 24199686, 22413962, 25200570, 27060930, 24186878, 24026211, 23604551, 24965700, 25805187 and 25583754.

<u>Post Evaluation Survey</u>

Demographic questions

	*FREE TEXT
Specialty	
Years of practice since medical school graduation	

**Please indicate how much % of your time do you typically dedicate to each of the following activities:**

	Less than 25%	25 to 50 %	50 to 75%	More than 75%
Clinical practice				
Research				
Clinical teaching				

Please rate your experience according to the items below

CLINICAL

	Little experience				Strong experience
	1	2	3	4	5
Dealing with patients in the same clinical domain of the narrative abstracts case vignette					
Dealing with cases with similar clinical complexity as in the case presented in the narrative abstracts vignette					
Dealing with patients in the clinical domain of the interactive visual display case vignette					
Dealing with cases with similar clinical complexity as in the case presented in the interactive visual display vignette					

TECHNICAL

	Little experience				Strong experience
	1	2	3	4	5
Experience in using computers for work activities					
Experience in using medical literature search tools in general (e.g., PubMed, UpToDate)					
Experience in using PubMed for medical literature search					

I would use the interactive visual displays to:									
	Strongly disagree				Neutral				Strongly agree
	1	2	3	4	5	6	7	8	9
help me with clinical decisions for specific patients									
find evidence during patient consultations									
find evidence after patient consultations									
prepare for patient appointments									
prepare for patient rounds									
prepare for teaching									
**Learnability**									
	Strongly disagree				Neutral				Strongly agree
	1	2	3	4	5	6	7	8	9
I was able to quickly learn how to use the user interface of interactive visual displays									

When comparing the two formats used in the study, I was able to:									
	4	3	2	1	0	1	2	3	4
	Narrative abstracts are much better				Neutral				Interactive visual display is much better
Scan the information quickly									
Comprehend the meaning well of the information presented									
Spend the least degree of mental effort									
Quickly obtain the gist of the study findings									
Be satisfied with the presentation (i.e., format of the display) of the information									
Locate information rapidly									
Identify relevant information to understand the study									
Effectively identify relevant RCTs from the search results									
Interpret individual RCT results quickly									
Accomplish task effortlessly									
Accomplish tasks with minimal frustration									
Easily use the user interface									
Quickly compare the results of multiple RCTs									
Quickly determine study relevance for the case vignette									
Enjoy exploring information									
Have fun seeking information to find answers									
Overall, which tool (e.g., PubMed or interactive visual display) would you prefer to use to help patient care decisions?									

Open comments

	***FREE TEXT**
Please give your suggestions for improving the interactive visual displays	
